# ‘I fear my partner will abandon me’: the intersection of late initiation of antenatal care in pregnancy and poor ART adherence among women living with HIV in South Africa and Uganda

**DOI:** 10.1186/s12884-022-04896-5

**Published:** 2022-07-15

**Authors:** Yussif Alhassan, Adelline Twimukye, Thokozile Malaba, Landon Myer, Catriona Waitt, Mohammed Lamorde, Angela Colbers, Helen Reynolds, Saye Khoo, Miriam Taegtmeyer

**Affiliations:** 1 Department of International Public Health, Liverpool School of Tropical Medicine, Pembroke Place, L3 5QA Liverpool, UK; 2 Infectious Diseases Institute, Makerere University, Kampala, Uganda; 3 School of Public Health and Family Medicine, University of Cape Town, Cape Town, South Africa; 4 Institute of Systems, Molecular and Integrative Biology, University of Liverpool, Liverpool, UK; 5 Radboud University Nijmegen Medical Centre, Radboud Institute for Health Sciences, Nijmegen, Netherlands; 6 Tropical Infectious Diseases Unit, Liverpool University Hospitals Foundation Trust, Liverpool, UK; 7 Department of International Public Health, Liverpool School of Tropical Medicine, Liverpool, UK

**Keywords:** Antenatal care, Antiretroviral therapy, Adherence to antiretroviral therapy, Dolutegravir, Antenatal care initiation, Booking, Women living with HIV, Uganda, South Africa

## Abstract

**Background:**

Many women in sub-Saharan Africa initiate antenatal care (ANC) late in pregnancy, undermining optimal prevention of mother-to-child-transmission (PMTCT) of HIV. Questions remain about whether and how late initiation of ANC in pregnancy is related to adherence to antiretroviral therapy (ART) in the era of national dolutegravir roll-out.

**Methods:**

This study employed a qualitative design involving individual interviews and focus group discussions conducted between August 2018 and March 2019. We interviewed 37 pregnant and lactating women living with HIV selected purposively for early or late presentation to ANC from poor urban communities in South Africa and Uganda. Additionally, we carried out seven focused group discussions involving 67 participants in both countries. Data were analysed thematically in NVivo12.

**Results:**

Women described common underlying factors influencing both late ANC initiation and poor ART adherence in South Africa and Uganda. These included poverty and time constraints; inadequate health knowledge; perceived low health risk; stigma of HIV in pregnancy; lack of disclosure; and negative provider attitudes. Most late ANC presenters reported relationship problems, lack of autonomy and the limited ability to dialogue with their partners to influence household decisions on health and resource allocation. Perception of poor privacy and confidentiality in maternity clinics was rife among women in both study settings and compounded risks associated with early disclosure of pregnancy and HIV. Women who initiated ANC late and were then diagnosed with HIV appeared to be more susceptible to poor ART adherence. They were often reprimanded by health workers for presenting late which hampered their participation in treatment counselling and festered provider mistrust and subsequent disengagement in care. Positive HIV diagnosis in late pregnancy complicated women’s ability to disclose their status to significant others which deprived them of essential social support for treatment adherence. Further, it appeared to adversely affect women’s mental health and treatment knowledge and self-efficacy.

**Conclusions:**

We found clear links between late initiation of ANC and the potential for poor adherence to ART based on common structural barriers shaping both health seeking behaviours, and the adverse impact of late HIV diagnosis on women’s mental health and treatment knowledge and efficacy. Women who present late are a potential target group for better access to antiretrovirals that are easy to take and decrease viral load rapidly, and counselling support with adherence and partner disclosure. A combination of strengthened health literacy, economic empowerment, improved privacy and patient-provider relationships as well as community interventions that tackle inimical cultural practices on pregnancy and unfair gender norms may be required.

**Supplementary Information:**

The online version contains supplementary material available at 10.1186/s12884-022-04896-5.

## Background

The use of antiretroviral therapy (ART) by pregnant women living with HIV (WLHIV) remains a key intervention for the prevention of mother-to-child-transmission (PMTCT). The effectiveness of ART in reducing vertical transmission and improving maternal health hinges on early initiation and maintenance of high levels of adherence in pregnancy and during breastfeeding [1]. Antenatal care (ANC) in the first trimester of pregnancy allows for timely initiation of treatment and a longer duration of ART exposure before delivery, a key determinant of mother-to-child transmission (MTCT) of HIV [2]. In a retrospective cohort study, Black et al. found more than seven weeks of ART exposure before delivery reduced MTCT rate to 0.3% and early ANC attendance was associated with good ART adherence postpartum [3]. Phillips et al. investigated the predictors of disengagement from ART postpartum among WLHIV in South Africa and reported a 4% increased risk for every additional gestational age week at ART initiation. They suggested ART adherence issues indicate wider issues in health seeking behaviour among women who present late for ANC that require further investigation [1].

Since 2012, Uganda and South Africa have adopted the WHO Option B + approach for PMTCT, recommending lifelong triple ART for all pregnant and breastfeeding WLHIV, regardless of CD4-cell count and clinical stage. Dolutegravir (DTG) has been introduced to replace efavirenz (EFV) as the preferred first-line regimen [4, 5]. ART services have been integrated into ANC and pregnant women are expected to be tested for HIV and initiated on ART if they are HIV positive on their first ANC visit. In South Africa, in line with the WHO recommendations on ANC [6], the Department of Health guidelines recommend eight ANC contacts for all pregnant women, with the first visit (‘booking’) occurring in the first 12 weeks of gestation [7, 8]. In Uganda, four ANC visits are recommended for uncomplicated pregnancies, with the first booking expected between 10 and 20 weeks of gestation [9]. Despite both countries achieving near universal coverage of at least one ANC visit, most pregnant women still present late. In Uganda, over 97% of pregnant women attended ANC at least once but only 29% made their first visit before the fourth month of pregnancy in 2016 [10]; final vertical transmission rate was nearly 6% in 2019 [11]. In South Africa, 94% of pregnant women presented for ANC in 2016, only 32% made their first visit before the fifth month of pregnancy, and 2% failed to access care until the eighth month [12]; MTCT rate was 3.3% in 2019 [13].While the introduction of dolutegravir with its rapid viral suppression is vital to improving health outcomes of HIV positive mothers who present for ANC late in pregnancy, adherence to ART treatment is central to maximising the effectiveness of dolutegravir for PMTCT. Therefore, understanding the relationship between late ANC attendance and ART adherence is essential to ensuring appropriate measures are adopted in national guidelines to realise the full benefits of dolutegravir rollout.

In sub-Saharan Africa a range of personal, healthcare system and socio-economic factors are associated with late ANC attendance [14] and poor ART adherence [15]. Our study countries reflect these findings. In Uganda, Kisuule et al. found that women presented for ANC late in pregnancy due to misperceptions about the purpose of early attendance and insufficient knowledge on the right gestation age to make the first visit [16]; Uldbjerg et al. identified facility HIV testing, attitudes of health workers, and a lack of partner support as the main barriers to early ANC initiation in Gulu District [17]; and non-adherence to ART during pregnancy was associated with inadequate counselling, fear of HIV disclosure, stigma and discrimination [18]. Similarly, in South Africa, Solarin and Black in inner-city Johannesburg discovered that, despite recognising the importance of ANC, most pregnant women booked late due to late recognition of pregnancy, lack of time, and provider practices such as asking women to return later [19]; and Myer and Harrison found most pregnant women in rural Hlabisa district delayed booking because they did not perceive significant threats during pregnancy [20]. In terms of ART adherence, Mepham et al. discovered sub-optimal medication usage among pregnant women in KwaZulu-Natal to be associated with widespread domestic violence, poverty, stigma and issues with partner disclosure [21]. In addition to HIV-related stigma, lifestyle behaviours and ART side-effects have been discovered as major causes of medication non-adherence among pregnant women in the Eastern Cape of South Africa [22].

Gaps remain in our understanding of the socio-cultural and relational aspects of women’s health seeking behaviour during pregnancy [23]. Few attempts have been made to understand how identified explanatory variables are related to each other and how they may be connected to the broader socio-economic and cultural factors in the study context. Thus, while there appears to be an overlap between the reasons for late ANC attendance and non-adherence, this relationship is poorly understood as are the contextual factors that determine women’s prenatal health seeking behaviour [1].

The purpose of this study was to explore whether and how late initiation of ANC in pregnancy is related to poor ART adherence in the era of national dolutegravir roll-out in South Africa and Uganda, including the deeper structural factors that underpin these behaviours. We sought to shift the locus of the enquiry from their usual client-population behaviour-based approach to the distal socio-cultural and institutional arrangements of women’s healthcare seeking behaviour.

## Methods

This study forms part of a larger research programme (DolPHIN-2) which aimed at increasing the evidence base for improving PMTCT, including a randomised controlled trial (NCT03249181) to evaluate the efficacy and safety of dolutegravir-based ART regimen initiated in late pregnancy [24]. As the success of PMTCT is dependent on optimal utilization of ANC and ART, exploring the relationship between the timing of ANC initiation and ART adherence in pregnancy was deemed to be crucial to DolPHIN-2. In South Africa, the DolPHIN-2 study site was in Gugulethu, a peri-urban township in Western Cape with approximately 98,500 people [25]. The area is economically deprived with an estimated HIV prevalence among women of 18.9% in 2015 [26]. In Uganda, DolPHIN-2 recruited from poor urban communities in and around Kampala, with HIV prevalence of around 6.9% [27]. In both countries, ANC and HIV treatment are public sector services delivered in primary health facilities free of charge.

### Study design

We used a descriptive qualitative design because it provides the most potential for exploring participant perspectives and the context that the views are embedded [28]. The study was underpinned by a constructivist paradigm and a phenomenological research approach. These emphasise multiple realities and truths, and the centrality of individual subjective experiences and meanings to understanding social phenomenon [28]. Thus, we explored the lived experiences and perspectives of pregnant and lactating WLHIV to gain deeper insights into the research questions: What are the main structural factors that shape delayed ANC attendance and non-adherence during pregnancy among WLHIV in South Africa and Uganda? How are these health seeking behaviours related? The study was informed by the socio-ecological model; this emphasises the interrelationships between individuals’ interpersonal, social, institutional and policy environments in shaping their health seeking behaviour [29], and thus provides a comprehensive framework for examining the range of factors influencing ANC attendance and ART adherence. We used ‘adherence’ to mean both adherence to treatment (pill taking) and attendance at clinic appointments [30].

### Study population and recruitment

A purposive sampling approach was applied to select participants, based on their experience of ANC attendance and HIV positive status, ensuring that diverse and useful data are obtained to adequately address the research questions [28]. To improve the reliability of the study findings, we used data from women who had utilised ANC and ART during pregnancy. We selected women who were pregnant or recent mothers (mothers of infants ≤ 6 months) living with HIV and 18 years and older, an age criterion that was necessary to overcome ethical challenges associated with research with minors. Participants included ‘early bookers’ and ‘late bookers’. While the focus of the study is on late bookers early bookers were included to compare the experiences and behaviour of late bookers. Based on national ANC guidelines, we classified a participant as a late booker in South Africa if she made her first ANC visit after 12 weeks of gestation [7], and in Uganda if it was after 20 weeks [9]. Early bookers were those who presented within the 12- and 20-week gestation periods in South Africa and Uganda respectively. Most of the participants were on an efavirenz (EFV)-based standard regimen, although several of the late bookers were DoLPHIN2 trial participants who had been initiated on a dolutegravir-based ART regimen. HIV status, type of regimen used and gestational age at booking were determined through self-reporting by the participant.

 Participants in both countries were approached at DolPHIN-2 affiliated health facilities: the Infectious Disease Institute and Kasangati Health Centre in Uganda and Gugulethu Maternity and Obstetric Unit in South Africa. Sample size for both the interviews and FGDs was determined by data saturation, deemed to have been reached when no new themes emerged at daily debriefing meetings with the research teams [31].

### Data collection

 Due to sensitivities around pregnancy and HIV in the study communities, individual interviews were employed to provide participants with privacy to speak openly. These were complemented with focus group discussions (FGDs) to stimulate fruitful discussion, allow a wide range of views to be explored and triangulate data [32]. Both methods were carried out in parallel to allow emerging findings from the interviews to be validated in subsequent FGDs. In each site, data were collected by four experienced female research assistants (RAs) between August 2018 and March 2019. The RAs were trained in the study protocol, research ethics and had extensive local knowledge.

 FGDs with 6–12 participants had one RA moderating and one taking notes. Data collection was conducted in Xhosa in South Africa and Luganda in Uganda in safe and private locations within facilities (individual interviews) and community centres (FGDs). Topic guides for both the individual interviews and FGDs were developed with broad open-ended questions covering topics on ANC initiation and ART adherence, including health seeking during pregnancy; reasons for presenting early/late; barriers and challenges to ART adherence in pregnancy; and intra-household partner relationship and decision-making during pregnancy (Additional file 1). Questions were piloted and revised iteratively as data collection and debriefings evolved.

### Data analysis

Audio recordings were transcribed verbatim and translated into English. RAs reviewed all transcripts for accuracy and completeness. Data were analysed in NVivo 12 software based on a framework analysis approach [33]. A common coding framework was inductively developed to facilitate inter-country comparison: 10% of the transcripts from each context was initially reviewed by two researchers (YA and AT) to develop independent coding lists; these were compared and merged; consensus was sought for variations in coding [34]. The framework was developed with flexibility to accommodate emergent new themes as coding evolved. Using the framework, each transcript was read for recurrent ideas; codes were assigned to relevant segments of the text; similar codes were aggregated to form themes that were then used to address the research questions [35]. Contradictory data identified during the analysis were initially treated as potential different viewpoints; they were subjected to further analysis to validate or refute them through detailed review of relevant transcripts/data and discussion among the research team; validated data were used to enrich insights on the issue [32]. To ensure rigor and trustworthiness, transcripts were independently coded, compared and discussed [36]. Data from individual interviews and FGDs were compared to triangulate the findings [37]. Emerging findings were discussed among the authors in regular meetings.

## Results

A total of 104 pregnant and lactating WLHIV participated across the two sites: 37 in individual interviews and 67 in FGDs (Tables 1 and 2). Most of the participants involved in the individual interviews (97.7%) were multiparous, with parity ranging from 1 to 5 children. The sample included similar proportions of single and married women. Most were of low educational status with little access to cash and unemployed or engaged in menial jobs such as farming, petty trading (in Uganda) and cleaning and farm labour (in South Africa). Many of the participants from South Africa relied on government social grants. Just under a third of the participants had booked early for ANC. Mean gestational age at booking was 8 weeks for the early bookers and 24 weeks for late bookers across both countries.

**Table 1:** Overview of the characteristics of participant for the individual interviews

Socio-demographic characteristics	South Africa	Uganda	Total
**Individual interviews (*n*** **= 37)**	***n*** **= 19**	***n*** **= 18**	N (%)
**Age**			
18–24 years	2	2	4 (10.8)
> 24 years	17	16	33 (89.2)
**Marital status**			
Married/living with partner	6	12	18 (48.6)
Single	13	6	19 (51.4)
**Educational level**			
Primary or no education	5	8	13 (35.1)
Secondary	14	9	23 (62.2)
Post-secondary	0	1	1 (2.7)
**Booking**			
Early	6	5	11 (29.7)
Late	13	13	26 (70.1)
**Maternity**			
Pregnant	13	8	21 (56.8)
Breastfeeding	6	10	16 (43.2)
**Parity**			
Primiparous	6	3	9 (24.3)
Multiparous	13	15	28 (75.7)
**Regimen used**			
DTG	8	6	14 (37.8)
EFV	11	12	23 (62.1)

**Table 2:** Overview of the characteristics of participant for the focused group discussions

Socio-demographic characteristics	South Africa (4 FGDs)	Uganda (4 FGDs)	Total
**Age**			
18–24 years	11	9	20 (29.9)
> 24 years	29	18	47 (70.1)
**Maternity**			
Pregnant	23	16	39 (58.2)
Breastfeeding	17	11	28 (41.8)

Important themes on common underlying structural factors shaping ART and ANC seeking behaviours and the direct links between late booking and ART adherence are described below along with illustrative quotes from the data.

### Common reasons cited for late ANC initiation and poor ART adherence

These are grouped into four adapted levels of the socio-ecological model: individual, interpersonal, facility, and community.

### Individual level

#### Poverty and lack of time

The health seeking behaviour of late bookers was hampered by time and financial constraints to access care. While ART and ANC services were supposed to be free, participants noted substantial indirect costs, including transport and food. In Uganda, they reported that women were often asked to pay for medical consumables and other ‘free’ services, including tipping/bribing workers for quicker service. As a result, many participants believed they needed to have money before they could attend ANC.

*“You can go to hospital, and they tell you they want gloves, polythene bags …, yet you do not have a single shilling.… you end up saying why go to hospital if they are asking for these things.”* (Late booker207, Uganda)

Further, many late bookers in Uganda reported food insecurity linked with their financial hardships as a barrier to medication adherence. Most associated ARV side effects with taking the drugs on an empty stomach and delayed or skipped them when they did not have food. They perceived it was unsafe for a pregnant woman to take ARVs without eating as that could harm the baby.

*“the challenge I have faced is [lack of] food. The medicine is very strong and if you take it without eating it will cause problem to the baby.…when the food is not ready, I wait until I have food* (Late booker 216, EFV user, Uganda)

Several participants in both countries reported difficulty juggling ANC/ART clinic visits with work and household chores. They reported being overwhelmed by household chores combined with the stresses of pregnancy, which interfered with their self-care, pill-taking and ability to fetch drugs from the clinic. In South Africa, several women were unable to negotiate time off from their low paid jobs for ANC and ART appointments.

*“When you’re pregnant you always have a lot of work to do. You wake up and you have to go out and find what to eat, do the washing, cleaning and cooking. Even when you are pregnant you still must do all the work, no help. You are tired and you even forget to take your drugs.”* (Late booker105, DTG user, South Africa).

In contrast to the early bookers, late bookers reported very little financial support from their male partners which hindered clinic attendance. Many reported being “too much dependant on their partners” and often “weakened by the HIV diagnosis to influence household decisions and to demand money and time for healthcare”. FGD participants in both countries noted men’s reluctance to financially support spouses to access ANC was related to mistrust e.g., when he suspected the paternity of the baby.

### Deficient health knowledge

Appropriate knowledge in reproductive health, antenatal care and PMTCT is critical to women booking early and optimal ART adherence, however these were mostly lacking among late bookers which undermined their health seeking behaviour in pregnancy. In both South Africa and Uganda, several late bookers reported a lack of awareness about the benefits of initiating ANC early in minimising MTCT and the right gestational age for first visits. Participants across both sites reported mixed messages from healthcare workers in relation to timing for ANC booking.

*“Most of us don’t know when the right time is to visit. Some say that you have to go immediately you know that you are pregnant. But sometimes you go there in the first month and …they tell you that you’re too early, that you are showing off. … so we are confused.”* (FGD 201, P8, Uganda)

In both settings, awareness of the potential benefit of early ANC initiation in minimising risk of MTCT was low among women prior to HIV positive diagnosis despite some having used ANC in previous pregnancies. Despite pregnant WLHIV receiving ART education during treatment initiation, many late bookers, reported lacking the confidence to recognise and respond appropriately to side effects from their medication. Several late bookers, especially those using EFV-based regimens, reported experiencing side effects which caused them to miss doses or delay taking drugs because they were unsure about how to deal with those effects. Perception of risk of ART drugs to the baby were widespread in both countries, and often prompted temporary suspension of treatment among these respondents.

*“I was told about the side effects at the clinic. … But when I started getting them, I was not sure if it was the drug. … I didn’t want to ask anybody [for confirmation]. …I was worried about the baby, so I stopped [taking the drugs] for a few weeks….”* (Late booker 121, EFV user, South Africa).

Several late bookers reported limited knowledge of pregnancy, which often led to delay in recognising their pregnancy and subsequent delay presentation for ANC. Some said they misinterpreted their pregnancy symptoms for the effects of ARVs or contraceptives. Although pregnancy tests were supposed to be free in public health facilities in both countries, frequent stockouts and long distances to facilities meant most women were having to resort to private outlets/clinics which they could not afford.

*“I got pregnant without knowing, and I spent 3 months feeling a bit unusual. The medicine [ARV drug] I used to swallow first made me to start vomiting. So, I thought it was that one... It was my first pregnancy. … I will say the health workers should teach the young ones about the symptoms of pregnancy….”* (Late booker 203, Uganda)

Participants in FGDs across both countries noted the patient education in maternity clinics were mostly didactic which often inhibited women’s ability to engage well to obtain the information they need about their care. Information about PMTCT were mostly targeted only at women who have been diagnosed with HIV, thus depriving potential mothers’ vital information to motivate them to present early for ANC and adhere to treatment when diagnosed with HIV.

### Perceived low health risks

Linked to the deficient health knowledge was the widespread belief among late bookers that ANC and PMTCT was only necessary in high-risk pregnancies. As a result, some WLHIV booked late for ANC because they felt well in the early stages of their pregnancy. Women who had previously experienced poor obstetric outcomes perceived a heightened sense of risk and were likely to prioritise ANC booking and treatment adherence when compared to those who had previous healthy pregnancies and uncomplicated childbirth.

“*I have 5 children and I delivered them very well, so that caused me to go late, I was thinking that “after all I deliver very well, and I am not ill”. … If I had known that I have the virus I would have come earlier.”* (Late booker 211, Uganda)

While HIV positive status was widely perceived as high risk in pregnancy, most late bookers reported only finding out about their HIV status at their first ANC visit late in pregnancy. FGD participants in both countries lamented over the lack of HIV testing opportunities in their communities. Many said they feared testing at the facility during ANC initiation due to potential inadvertent disclosure, especially as they may be accompanied by partners. They suggested HIV testing is decoupled from ANC booking, and for opportunities to be created to allow women to test in private.

### Interpersonal level

#### Relationship insecurity and limited autonomy and control over household decisions

The nature of women’s relationship with their partners emerged as an important determinant of their health seeking behaviour during pregnancy. Compared with the early bookers, several late bookers in Uganda and South Africa exhibited profound relationship insecurity, characterised by mistrust, neglect and fear of breakup. Many had experienced a disrupted first marriage, having separated, and remarried or cohabiting. Some were living in a polygamous relationship, either sharing the same household with other wives, or separately in another house. Partly due to these living arrangements, most late bookers reported having limited opportunity to dialogue with partners about their health, including making reproductive plans and negotiating resources for care. As a result, pregnancies were mostly unplanned, and it took long to decide whether to keep the pregnancy, and to initiate ANC. Suspicion of infidelity, domestic violence and neglect were commonly noted among the late bookers, which undermined their healthcare seeking during pregnancy.

*“We have issues. We are not married, and he has another wife whom he lives with. We don’t see each other every day, and don’t get the chance to talk often. If we were living together at least he would have encouraged me to go and book or to take my medicine…. We fight a lot because he thinks I’m cheating on him.”* (Late booker 115, South Africa)

In contrast, most early bookers talked about stable relationships, and reported a high degree of intra-household cooperation, planning, and support from partners. This socio-cultural characterisation appeared to overlap with the pattern of ART adherence among women across both countries, with women in stable relationships reporting high level of adherence.

*“It [pregnancy] was planned. … we were always buying the pregnancy test to check. As soon as I found that I was pregnant my partner told me to go to the clinic, he was very supportive…”* (Early booker 202, Uganda)

#### Lack of disclosure of pregnancy and HIV status

Across both settings, women’s lack of disclosure of their pregnancy and/or positive sero-status to their partners was identified as a major barrier to optimal health seeking during pregnancy. Non-disclosure of pregnancy was mostly common among women in unstable relationships who were often unsure about whether the pregnancy would be desired by the man or not. This deprived women of the support and motivation needed from their partner to seek ANC and ART care.

*“I didn’t tell him about the pregnancy until it started showing. …I didn’t know how he will react. He always said he was not ready. …because of that I was also reluctant, and nobody pushed me to go. That is why I booked late.”* (Late booker 111, South Africa)

Treatment adherence was impacted by partner non-disclosure of HIV positive status. Most women who were diagnosed late in pregnancy said they delayed informing their spouses; others hoped they could keep their status secret indefinitely. This compounded difficulty in coping with their diagnosis and hampered adherence. Several reported they missed a dose or took medication late because their partner was around.

*“ I had not disclosed [to my husband] for one month … [because] I didn’t know how he will react. It was hard because nobody was there to console me and encourage me to take my drugs…. At the beginning whenever we were all around at home, I will not take my pills…”* (Late booker 216, Uganda)

HIV status disclosure was particularly challenging among late bookers in fragile relationships who often feared being accused of infidelity and/or a breakup. Concerns about the baby getting infected with HIV due to late diagnosis intensified the difficulty for late bookers to disclose. Many lamented over their financial challenges and noted they could not cope if the man abandoned them.

*“… I’m afraid to tell him, already he said he does not trust me. He will think I am sleeping with other men [if I disclose], and he will leave me. He will think it is too late and that the baby will get it [HIV] and he will be upset …I don’t have a job. …I have two other children from my first husband. I can’t cope with all these children if he left me.”* (Late booker 216, Uganda)

Women who had not disclosed their HIV positive status were less likely to receive support from their partners with their health, including HIV treatment to minimise the risk of vertical transmission. In contrast to the late bookers, many of the early bookers were women who were aware of their HIV positive status prior to pregnancy, had informed their partners about their status, and a joint decision made for her to initiate ANC early and attend ART appointment regularly to ensure the safety of the baby.

### Facility level

#### Lack of privacy and confidentiality

Participants across both sites complained about lack of privacy in healthcare facilities and worried sensitive information about their pregnancy or HIV status may be compromised. Both healthcare workers and patients were perceived as potential sources of breached confidentiality. Some WLHIV were concerned that regular facility visits, as required of new HIV patients, increased the risk of inadvertent disclosure. HIV care was particularly challenging for women when they were no longer able to use antenatal and postnatal facility visits as a cover.

*“The one thing which makes us not go to the clinic early is the fear that you will meet a nurse that you know. …. You’re afraid that she will go and talk about you in the community. …[also]* w*hen you’re at the clinic receiving counselling, other patients will be watching and listening*.” (Late booker206, Uganda)

### Negative provider attitudes

Disrespect and mistreatment by healthcare workers were widely reported in both countries as barriers to early initiation of ANC use of ART. Late presenting women were particularly susceptible to negative provider relationships as they were rebuked for booking late, which discouraged them from making subsequent ANC and ART appointments.

*“they talk anyhow to you without caring how you will feel. You are shouted at… We also don’t want that.….”* (Late booker113, South Africa)

*“Some of the health workers are very rude.... They ignore you and leave you to stand in the sun. It is very bad when you are pregnant.… remember some of us are already depressed from being told we have HIV; you are not sure if you will live.”* (Late booker292, Uganda)

Late bookers were noted to be more likely to present attributes leading to reprimand by healthcare workers, including high parity and short birth spacing. In Uganda, some perceived that they experienced negative provider attitudes due to their inability to pay a tip/bribe. Several said they were rebuked and discriminated for using traditional or herbal medicine. Many participants in both countries reported fearing mistreatment if presenting without their partner as they knew this was recommended.

*“Some of the health workers if your appearance is not appealing [ look poor], they just ignore you. If they see you cannot give a tip, they ignore you. … when your partner doesn’t want to go with you... they will not treat you well if he is not there.”* (FGD 292, P4, Uganda)

### Long delays and unsuitable opening hours

In both settings, a typical visit to the clinic for ANC or ART lasted about a half to a full day which made it difficult for pregnant women to juggle responsibilities. A lack of suitable resting and toilet facilities at clinics coupled with being pregnant made attendance stressful.

*“… when we go for ANC, the queues are long most times and we women get fed up very fast, sitting for hours you feel like you are dying. Most times we stand up the whole time, no proper place to sit and even a place to ease yourself.”* (FGD 291, P10, Uganda).

Due to the inconvenience of clinic attendance, several participants said they wanted to reduce the number of times they visited and booked late. The opening times for ANC, mostly limited to a few days of the week, were found to be constraining. In South Africa, a few participants expressed security concerns over them needing to travel early in the morning to queue for ANC or ART.

### Community level

#### Stigma of HIV in pregnancy

FGD participants across both countries identified pervasive stigma about HIV in pregnancy. They noted widespread belief that pregnant HIV positive women would automatically infect their baby with the virus. Such community perceptions were found to promote stigma and discrimination against HIV positive women and undermined disclosure and utilisation of ART and ANC services. As this interview participant reported.

*“People in the community think that once you are HIV positive you will give birth to an HIV positive baby. Some see us and say why do we get pregnant, that we are going to give birth to orphans. It is not a good feeling, so you try to avoid people knowing.”* (Late booker 108, South Africa)

Perception about HIV being associated with promiscuity and infidelity was widespread and led to HIV expectant mothers fearing that the paternity of their baby might be questioned.

At the community level, participants noted a general lack of adequate knowledge about PMTCT, particularly the benefits of dolutegravir in rapid viral suppression to avert MTCT, which perpetuated the stigma against pregnant HIV positive women.

“*Most people still don’t know that with ART you can give birth to an HIV negative child. They need to sensitise everybody in the community to understand what ART can do.”* (Uganda, FGD 202, P5)

#### Belief in evil spirits and use of traditional medicine

Belief in supernatural threats to health was widespread in both countries, particularly among late bookers. Many were reluctant to disclose their pregnancy early, and initiated ANC late, because they feared detection by ‘evil eyes’ would cause miscarriage. Early ANC initiation and pregnancy disclosure was seen as risking bewitchment from other women, especially jealous co-wives.

*“We never want to admit to being pregnant because you are afraid people will bewitch you, so you end up not coming for ANC because people will see you. Even when people compliment your pregnancy glow, you become defensive because you are afraid people will kill your unborn baby.”* (FGD 193, P6, South Africa)

In South Africa, pregnant women were believed to be susceptible to attack by evil spirits if they went out at night, which discouraged many from ANC and ART attendance.

*“In our culture women are not supposed to go out in darkness when pregnant otherwise they can be attacked by bad spirits. So, it is a challenge because you have to leave home early in the morning when it is still dark to queue at the clinic...”* (FGD 192, P3, South Africa)

Compared with early bookers, most late bookers often resorted to traditional/spiritual healers for treatment/protection on illnesses believed to be caused by evil spirits. Some said they resorted to herbal medicine and healing from spiritual churches as a substitute for ART. The use of traditional medicine was often reprimanded by health workers, which in turn discouraged many from accessing ANC and ART.

*“When we put these strings around our waist, the nurses in the clinics swear at us a lot. …even though we are just protecting ourselves. Because of that you don’t want to go to the clinic ….”* (FGD 192, P1, South Africa)

### Effect of late booking on ART adherence

Several direct links between late initiation of ANC and poor ART adherence were observed in the data. Participants with previously unknown HIV status were mostly distraught by the news of their positive diagnosis late in pregnancy which often affected their mental health and treatment self-efficacy. Part of their anxiety was mostly related to concerns about the baby becoming infected. Many expressed despair for diagnosing late and believed their baby will be infected.

*“When they told me I was positive, I did not believe it. I was shocked, I lost interest in everything. I went days without eating.…the doctor said because I was late the baby will get it …. I wanted to even abort the pregnancy, but it was too late. When I got home, I did not take the medicine they game me because I had lost interest.… now the baby is also positive.”* (Late booker 209, Uganda)

Some late women reported suicidal ideation due to their late diagnosis; a few said they resorted to excessive drinking of alcohol; while several others said they were “so depressed that [they] lost the motivation to follow through on their treatment”.

Further, the delay diagnosis appeared to add another layer of complication to disclosure due to women perceived increased risk of MTCT, as this FGD participant noted: “*Disclosing your HIV positive status to the man during pregnancy is hard enough let alone when he thinks the child will definitely get it. … you do it at your own risk because he will run away.”* The lack of partner disclosure often deprived women the needed support for their treatment during pregnancy.

Many late bookers said they were often scolded by some health workers for presenting late for ANC. This, coupled with their HIV diagnosis, acted as significant stressors affecting their ability to comprehend treatment education during initiation.

*“They teach us at the clinic but when you have just been given the bad news and the health workers are blaming you, you can’t concentrate, there is nothing you will understand.”* (Late booker 117, South Africa)

While the availability of dolutegravir was reassuring for health care workers as a possible solution to late booking, women were not sufficiently informed about its benefits in rapid viral load reduction for its availability to impact decision-making about booking and adherence.

## Discussion

**Fig. 1:**
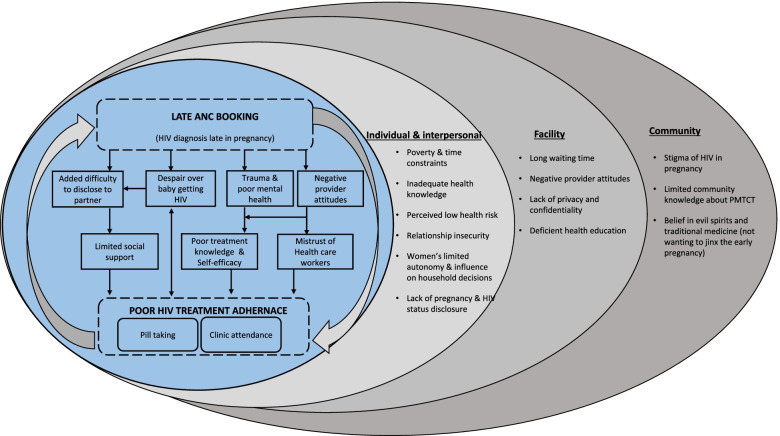
Relationship between late initiation of antenatal care and poor adherence to antiretroviral therapy among women living with HIV in Uganda and South Africa. Legend: Diagram depicts the overlap between late ANC initiation and poor ART adherence based on the socioecological model [29]. The innermost (blue) circle demonstrates the direct pathways through which late ANC booking and related HIV diagnosis late in pregnancy lead to poor ART adherence. The outer (grey) circles show common underlying factors at the individual, interpersonal, facility and community levels that shape health seeking behaviour (both ANC initiation and ART adherence) of HIV positive women during pregnancy

We found overlaps between the underlying structural determinants of late ANC initiation and poor ART adherence among WLHIV in both South Africa and Uganda. As depicted in the outer (grey) circles of Fig. 1, these common factors include poverty; women’s inadequate health knowledge; perceived low health risk; stigma of HIV in pregnancy; lack of pregnancy and HIV status disclosure; and negative provider attitudes. Relationship insecurity among women constrained their autonomy and control over household decisions and resources to support their health during pregnancy. Widespread perception of a lack of privacy and confidentiality in maternity and ART clinics compounded the additive risks associated with early disclosure of pregnancy and HIV. Several direct links between late HIV diagnosis and poor ART adherence were observed, as noted in the innermost (blue) circle of Fig. 1. Women were likely to be subjected to negative provider attitude for presenting late which hampered participation in treatment counselling and festered provider mistrust and subsequent disengagement. HIV diagnosis late in pregnancy affected women’s mental health and treatment knowledge and self-efficacy. It complicated women’s ability to disclose their HIV status to significant others and therefore deprived them of essential social support for treatment adherence. During ANC booking providers could screen for late bookers, including women with markers of social vulnerability such as relationship problems and unwanted pregnancy and provide targeted support to improving ART-related self-efficacy, skills for safe partner disclosure, and mental health. Women who present late may be supported with better access to effective antiretrovirals, such as dolutegravir, that are easier to take and decrease viral load rapidly. This could break the cycle linked to despair of infected baby and improve the mental health resilience of late bookers.

Women in our study perceived the risks of early ANC to outweigh the benefits except among WLHIV where prior knowledge of HIV status appeared to be a strong catalyst for early ANC initiation to prevent MTCT. Therefore, expanding community HIV testing capacity, including home-based self-testing and mobile testing, could improve early recognition of HIV positive status and ANC initiation [38]. Improving women’s knowledge of reproductive health and access to pregnancy testing would enable them to recognise their pregnancy early and minimise the risk of unplanned pregnancies [39]. Our community findings showed women to be making decisions without being fully informed. Although health promotion campaigns may have contributed to the increased awareness and uptake of ANC among women [40], they do little to dispel (or work alongside) myths that evil spirits threaten early pregnancy. As a result, many women deemed traditional and spiritual intervention to be more suitable, and perhaps more convenient and confidential, than biomedical intervention, endorsing similar findings in Mozambique [23] and Pretoria [41]. Specific information on the benefits of early booking, the right gestational age for first ANC visit, the benefits of PMTCT and the pros and cons of different treatments was also lacking. With many women discovering their HIV status in pregnancy, messages about the benefits for PMTCT need to be targeted to everyone, not just WLHIV. Further, patient education in facilities may be hampered by a didactic pedagogy which limits women’s ability to ask questions and obtain relevant information. While informal networks have been widely identified as a key source of health information among women in resource-limited contexts [42], most newly diagnosed WLHIV were discouraged from leveraging these sources due to lack of disclosure. The high penetration of community health workers and the popularity and closeness to communities of peer networks in most African contexts make these ideal safe spaces for education on ANC and HIV care in Uganda and South Africa [43].

Women’s limited influence over decisions about their health and household resources was driven in part by financial and social insecurities that are reinforced by gender norms [44–46]. In patriarchal societies, such as those that our study was conducted, men exercise greater control over intra-household decisions over resource allocation as well as cultural norms which require women to undertake most household chores and be subservient to their male partners [47, 48]. Asymmetric relationships constrained women’s access to time and financial resources and their ability to dialogue with their partners over their healthcare needs (e.g. support with pills fetching, accompaniment on ANC visits) and reproductive plans [49]. Concerns about HIV related stigma and discrimination, which discouraged women from accessing ANC and ART, further demonstrate women’s limited agency both the household and community. In a context of a fragile relationship, women feared that a positive HIV status would aggravate the relationship and diminish their social standing and access to resources. Pregnancy tended to raise the stakes of relationship breakup for women, especially if she was unemployed. In an attempt to improve social capital, women may strongly commit to societal norms, including those around pregnancy that undermine early ANC attendance and delay pregnancy disclosure [50]. Traditional demand-side interventions to improve women’s use of ANC and ART adherence, predominantly based on awareness raising, education and financial incentives have been limited by their failure to address these structural barriers. Complementary measures are required that promote women’s agency and redress asymmetric power relations at households, community, and facility levels [51]. One approach has been to enhance women’s financial independence through increased participation in paid employment and income generating activities [47, 52]. Such measures need to be embedded within a broader programme that also works with men to improve intra-household decision-making, encourage participation in ANC and ART, and redress unfair gender norms in the division of resources and responsibilities within the household. Tolhurst et al. have cautioned that simply increasing women’s opportunities to earn independent income could lead to men reneging on their financial responsibilities, leading to women filling the gap and their inability to mobilise greater resources to break from male subordination [47]. Effective intra-household decision-making and communication is also crucial to mitigating potential risks of gender-based violence that are associated with women’s economic empowerment in patriarchal societies [53, 54]. Further, community-wide interventions are needed and should also consider engaging with traditional and faith-based healers considering their influence in women’s health seeking behaviours in pregnancy.

While perceived poor-quality service have been widely identified to undermine healthcare seeking in pregnancy in previous studies [50, 55, 56], we found that women with low socioeconomic attributes, including late bookers, are disproportionately affected. Poor women already experience higher opportunity costs associated with seeking care [18] and, in our study, appeared to be more susceptible to negative provider attitudes due to their perceived association with negative health seeking behaviours such as use of traditional/herbal medicine, high parity, low birth spacing and delayed ANC booking, which diminished their trust in the healthcare system and discouraged future use [57, 58]. In Uganda and South Africa, the introduction of dolutegravir based treatment, with its quick viral suppression [24], could reduce health worker anxiety of risks of MTCT, and subsequently improve pregnant women’s health seeking experiences in maternity clinics. We are yet to know if it will impact negatively on early ANC attendance as women may feel less compelled to book early. Healthcare workers should leverage and promote the positive message of dolutegravir in rapid viral suppression to reassure late presenting mothers and to build trust for improved engagement in ANC and ART. Further, it is crucial to streamline clinic operating systems to minimise waiting times and improve privacy and patient-provider relationship. As well as training health workers on patient confidentiality and customer service, current procedures for delivering ANC and HIV care in facilities would merit a review to identify and rectify areas that breach patient confidentiality. With many maternity clinics lacking adequate space, a pre-booking appointment system could minimise over-crowding and improve patient privacy and waiting time, as evidence from Mozambique suggests [59]. Such measures could further engender greater accountability and improve provider attitude towards patients.

## Limitations

The data on poor adherence among late bookers should be interpreted with some caution since the study was cross-sectional with the interviews conducted only once during pregnancy or postpartum. Variations in the attributes and health seeking behaviour between early bookers and late bookers reported were not based on statistical significance. Reported ART adherence among early bookers may have been confounded by some participants involvement in the DolPHIN-2 clinical trial who were given more extensive information and counselling than is normally the case in health facilities. Future research would benefit from longitudinal follow-up of late bookers to further ascertain how late booking influences poor adherence to make the findings more generalisable. The study is based on self-reports and asked about women’s history of healthcare seeking and may therefore be subject to recall bias. There is also a potential for social desirability bias and under-reporting of barriers among respondents who might have been hesitant to share their true beliefs and challenges with the data collectors. Most of the participants were older women; only a few adolescents, who are noted to be more susceptible to delayed booking and poor ART adherence, were included in the study sample. The different time points used to classify early and late booking in the two study settings may have affected inter-country comparison. However, our central objective was to explore wider health seeking behaviour of HIV positive women in pregnancy rather than what transpired within the different time points. The findings are based on a greater proportion of late bookers in the study sample which may have disadvantaged the views of early bookers, although emphasis on late bookers was necessary as they were the primary focus of the study.

## Conclusions

This study has demonstrated ways in which pregnant WLHIV negotiate healthcare seeking to understand the intersections of ANC booking and ART adherence. We have argued that late ANC attendance is associated with poor ART adherence and that both are both associated with women’s limited agency and lack of influence over household decisions, poverty, inadequate health knowledge, stigma of HIV in pregnancy, lack of disclosure, and negative provider interaction. Further, HIV diagnosis late in pregnancy impacts negatively on women’s ability to disclose their HIV to their partners, mental health and treatment knowledge and efficacy. While key alterations are needed in ANC and HIV services including improving privacy and provider-client interaction and targeted adherence support for late bookers, the complexity of the issues suggest that the solutions lie beyond the healthcare system alone. There is the need for measures to improve women’s personal capabilities for healthcare through health literacy and economic empowerment as well as community interventions to tackle inimical cultural norms on pregnancy, HIV and pregnancy-related stigma.

## Supplementary Information

**Additional file 1.**

## Data Availability

Data are available from the corresponding author on request.

## Abbreviations

ANC: Antenatal care
ART: Antiretroviral therapy
DTG: Dolutegravir
EFV: Efavirenz
FGD: Focus group discussion
HIV: Human immunodeficiency virus
MTCT: Mother-to-child transmission
PMTCT: Prevention of mother-to-child-transmission
RA: Research assistant
WLHIV: Women living with HIV
