# Supplementary material for: ‘I fear my partner will abandon me’: the intersection of late initiation of antenatal care in pregnancy and poor ART adherence among women living with HIV in South Africa and Uganda
Source: BMC Pregnancy Childbirth. 2022 Jul 15;22:566. doi: 10.1186/s12884-022-04896-5 (PMC9284724; doi:10.1186/s12884-022-04896-5)
Supplement: Supplementary file 1 — Additional file 1. [file 12884_2022_4896_MOESM1_ESM.doc]

**Additional file 1: Interview and focused group topic guides used in the study**

1. **Interview with pregnant & lactating HIV positive mothers**

**Knowledge and experience of ANC**

1. Please tell me about what you do for your health when pregnant?

- Probe use of traditional medicine
- Probe knowledge of antenatal care (including benefits, risks, frequency of visit, when first ANC visit should take place)

1. Tell me about your understanding of antenatal care initiation (or booking)

- Probe: what happens during the first ANC visit?
- Probe: what benefits do you associate with booking?
- Probe: what benefits or risks do you associate with early booking?
- Probe: what benefits or risks do you associate with late booking?
- Probe: at what stage of the pregnancy would you prefer to book? Why?

1. Tell me about your experience of booking for ANC during your current /recent pregnancy

- Probe: how old was your pregnancy when you booked? Why this time?
- Probe: how was the care you received during your booking visit?

**Barriers & enablers of early ANC booking**

1. *EARLY BOOKER*: What factors prompted or helped you to book earlier for ANC?
2. *LATE BOOKER:* what reasons made you to book later than usual?
3. Did you face any problems when you were booking for antenatal care? Please explain

- Probe problems related to healthcare providers
- Probe problems related to the household
- Probe problems related to the community

1. What would have made you to book earlier?

**HIV diagnosis & Disclosure**

1. Tell me about your experience of HIV diagnosis during pregnancy.

- Probe: How did you feel when you were informed about your positive results?
- Probe: would you have felt different if you were diagnosed earlier?
- Probe: how do you feel diagnosing later in pregnancy may have affected your treatment

1. How easy or difficult was it for you to inform your partner and other family members about your HIV positive status?

- Probe: would you have found disclosure differently if you had discovered your status earlier?
- Probe: how has non-disclosure affected your treatment?
- Probe: what additional support would you need to address the challenges caused by non-disclosure?

**Knowledge and experience of PMTCT**

1. Please tell me a bit about what you know about transmission of HIV from mother to babies (MTCT).

- Probe: how does MTCT make you feel?
- Probe: how does it affect your health seeking behaviour during pregnancy?

1. What do you know about the treatment/drugs used for preventing mother-to-child transmission of HIV?

- Probe: how do you feel about these drugs?
- Probe: what harm and benefits do you associate with this drugs/treatment?

1. Tell me about your experiences with starting ART

- Probe: when did you start, and why that time?
- Probe: did you face any challenges starting? Please explain
- Probe: what motivated you to start?
- Probe: were you happy with the level of information, counselling and support given to you by the health care workers?
- Probe: If not, what should have been done differently?
- Probe: when should be the ideal time to start ART in pregnancy?

1. What HIV drugs do/did you use when you were pregnant or breastfeeding?

- Probe: How do you feel about taking these drugs?
- Probe: How well are/were you taking your HIV drugs during pregnancy/breastfeeding?
- Probe: Have you had any problems using these drugs? Please explain
- Probe if ARVs are seen differently in terms of their benefits and side effects?

**ART adherence**

1. Tell me about your experience of adherence to ART while being during pregnancy or breastfeeding
2. There are certain ways people are told to take their HIV drugs (e.g., take a certain number of pills at a certain time of day, visit the clinic on certain days, etc), how has it been for you to follow these ways?
3. What makes it hard for you to follow the right treatment regime?

- Probe cost, transportation, childcare, stigma, non-disclosure, etc

1. What helps you to follow the right treatment regime?

- Probe use of pill boxes, daily routine, reminders, regular mealtimes, privacy, support of friends or family, etc

1. As a breast-feeding mother are there any other things that affect your adherence to ART?
2. What changes would you like to see in the way that HIV treatment is delivered to pregnant and breast-feeding mothers n?

- Probe: What additional support are needed to help women who present late in pregnancy with their HIV treatment?

1. **FGD with women living with HIV**

**Health seeking behaviour in pregnancy**

1. What kind of things do women in the community do for their health when they are pregnant?

- Probe women’s health seeking behaviour during pregnancy
- Probe use of traditional medicine, traditional birth attendants, etc

1. What common cultural beliefs and practices in the community are related to pregnancy?

- Probe how these beliefs and practices affect ANC attendance

1. How do people in the community (both women & women) perceive antenatal care?

- Probe: what benefits do they associate with ANC?
- Probe: what concerns do they have about ANC?

1. How are decisions about the use of antenatal care made in the family?

- Probe: what role does the man play?
- Probe: what role does the woman play?

**Reasons for late ANC initiation**

1. What reasons do women in the community have for not booking early for antenatal care?

- Probe factors related to healthcare providers
- Probe factors related to the household level
- Probe factors related to the community level
- Probe potential effects of cultural beliefs and practices
- Probe potential effects of pregnancy non-disclosure, stigma and intimate partner violence

1. What can be done differently to enable women to initiate ANC early?

**HIV diagnosis & disclosure**

1. How do people in the community perceive HIV in pregnancy?

- Probe stigma and discrimination related to HIV in pregnancy
- Probe perception of HIV diagnosis **late** in pregnancy

1. What challenges do women face when they are diagnosed with HIV in pregnancy?

- Probe challenges faced when diagnosis occur late in pregnancy

1. How easy or difficult is it for pregnant women to disclose their HIV positive status to their partner?

- Probe how late diagnosis may affect disclosure
- Probe how non-disclosure affects HIV treatment?
- Probe what additional support may be needed to address challenges of non-disclosure?

**Engagement in HIV treatment**

1. What benefits and risks do pregnant and breastfeeding women associate with ART?

- Probe about common side effects experienced and how this affect adherence

1. What problems do pregnant and breast-feeding women face with their HIV treatment?

- Probe potential effects of non-disclosure and stigma
- Probe potential effects of pregnancy and breastfeeding activities

1. What challenges do pregnant women who book late for antenatal care face with their HIV treatment?
2. What can be done to improve ART uptake and adherence among pregnant and breast-feeding women in the community?

- Probe additional support needed to help women who present late in pregnancy with their HIV treatment.
